# On the Optimal Combination of Elliptically Distributed Biomarkers to Improve Diagnostic Accuracy

**DOI:** 10.3390/genes15091145

**Published:** 2024-08-30

**Authors:** Shiqi Dong, Zhaohai Li, Yuanzhang Li, Aiyi Liu

**Affiliations:** 1 Department of Statistics, The George Washington University, Washington, DC 20052, USA; dsq0405@gmail.com (S.D.); zli@gwu.edu (Z.L.); 2 Department of Defense (Retired), Silver Spring, MD 20910, USA; yuanzhang.li@yahoo.com; 3 Eunice Kennedy Shriver National Institute of Child Health and Human Development, Bethesda, MD 20817, USA

**Keywords:** ROC curve, elliptical distribution, biomarkers, AUC, likelihood ratio combination, nonparametric maximum likelihood estimate

## Abstract

Diagnostic biomarkers play a critical role in biomedical research, particularly for the diagnosis and prediction of diseases, etc. To enhance diagnostic accuracy, extensive research about combining multiple biomarkers has been developed based on the multivariate normality, which is often not true in practice, as most biomarkers follow distributions that deviate from normality. While the likelihood ratio combination is recognized to be the optimal approach, it is complicated to calculate. To achieve a more accurate and effective combination of biomarkers, especially when these biomarkers deviate from normality, we propose using a receiver operating characteristic (ROC) curve methodology based on the optimal combination of elliptically distributed biomarkers. In this paper, we derive the ROC curve function for the elliptical likelihood ratio combination. Further, proceeding from the derived best combinations of biomarkers, we propose an efficient technique via nonparametric maximum likelihood estimate (NPMLE) to build empirical estimation. Simulation results show that the proposed elliptical combination method consistently provided better performance, demonstrating its robustness in handling various distribution types of biomarkers. We apply the proposed method to two real datasets: Autism/autism spectrum disorder (ASD) and neural tube defects (NTD). In both applications, the elliptical likelihood ratio combination improves the AUC value compared to the multivariate normal likelihood ratio combination and the best linear combination.

## 1. Introduction

Diagnostic biomarkers are essential in the diagnosis and prediction of diseases, as well as in clinical trials. Some well-known biomarkers include glycated haemoglobin (HbA1c) for diagnosis of diabetes [1], procalcitonin (PCT) and C-reactive protein (CRP) for COVID-19 [2,3], and prostate-specific-antigen (PSA) [4] for cancer. As new biomarkers for early disease detection and prevention continue to be developed, it is imperative to assess their accuracy in diagnosing diseases by comparing them with existing biomarkers.

Diagnostic accuracy indices are commonly used to measure the ability of a biomarker to discriminate or predict a disease. Consider a single biomarker *M*, which is measured on a continuous scale. A subject will be diagnosed as diseased if *M* is larger than a predefined cut point value *c*. Usually, it is assumed that subjects with higher biomarker values are more likely to be diseased. If this assumption does not hold, appropriate transformations can be applied. Corresponding to a cut point value *c*, the sensitivity (also called the true positive rate) of this biomarker is the proportion that a diseased person is correctly identified, and the specificity (also called the true negative rate) is the proportion of a non-diseased person that is correctly identified, that is,

(1) $$\left\{\begin{array}{c} s(c)=P(M>c\;|\;\;\mathrm{the}\;\;\mathrm{subject}\;\;\mathrm{is}\;\mathrm{diseased})\\ p(c)=P(M<c\;|\;\;\mathrm{the}\;\;\mathrm{subject}\;\;\mathrm{is}\;\;\mathrm{not}\;\;\mathrm{diseased}). \end{array}\right.$$

where s(c) is the sensitivity (TPR) and p(c) is the specificity (TPR).

The ROC curve is plotted by the sensitivity against the 1-specificity (false positive rate) over all possible cut point values of c∈R. Most measure indices, such as the area under the ROC curve (AUC), are derived from the ROC curve [5].

However, it has been widely acknowledged by medical researchers that diagnosis based on one single biomarker may not provide sufficient accuracy. To improve diagnostic performance, researchers have proposed numerous methods to combine multiple biomarkers and the corresponding measurements to help clinicians make better diagnostic judgments. Su and Liu (1993) derived the optimal linear combinations that could maximize AUC value [6]. They also proved that the best linear combination is equivalent to the optimal combination if both the case and control population follow multivariate normal distributions with the same covariance matrix. In 2000, Pepe and Thompson discussed an empirical estimation for solutions of the optimal linear combinations, which is non-parametric and robust against misspecified distributions [7]. However, the empirical estimation is limited by the sample size. In 2005, Liu et al. derived alternative linear combinations that exhibit higher sensitivity over a range of high (or low) specificity [8]. Furthermore, Pepe et al. (2006) proved that the likelihood ratio combination is the optimal combination [9]. The likelihood ratio, though optimal, is sensitive to distributional assumptions, which are often difficult to justify.

Although many research results have been derived based on multivariate normal distribution, this assumption is often too stringent. In clinical trials, data may follow distributions with heavy tails, such as the multivariate t distribution or light tails. In this paper, we aim to extend the optimal combination of biomarkers to a broader distribution family, elliptical distribution, to enhance its practical utility.

Let *X* be a *d*-dimensional random vector. *X* is said to be ‘elliptically distributed’, if and only if there exists a vector $\mu \in R^{d},$ a positive semidefinite matrix $\Sigma \in R^{d\times d},$ and a function $\phi :R_{+}\to R$ such that the characteristic function $t\mapsto \varphi_{X-\mu }\left(t\right)$ of X−μ corresponds to $t\mapsto \phi \left(t^{T}\Sigma t\right),t\in R^{d}$ [10].

For an elliptically distributed random vector *X*, denoted as $X∼E_{d}\left(\mu ,\Sigma ,\phi \right)$ where $\mu \in R^{d}$ is the location parameter and $\Sigma \in R^{d\times d}$ scale matrix, it can be represented stochastically by $X\overset{d}{=}\mu +R\Lambda U^{\left(d\right)}$ with $\Lambda \Lambda^{T}=\Sigma$, where $U^{\left(d\right)}$ is uniformly distributed on the unit hypersphere with d−1 topological dimensions, R is a non-negative random variable stochastically independent of $U^{\left(d\right)}$. Then, the p.d.f of X is given by

(2) $$f_{X}\left(x\right)=\sqrt{\det\left(\Sigma^{-1}\right)}\cdot g_{R}\left({\left(x-\mu \right)}^{T}\Sigma^{-1}\left(x-\mu \right)\right),\;x\neq \mu$$

where

$$g_{R}\left(t\right):=\frac{\Gamma \left(\frac{d}{2}\right)}{2\pi^{d/2}}\cdot {\sqrt{t}}^{-(d-1)}\cdot f_{R}\left(\sqrt{t}\right),\;t>0$$

and $f_{R}$ is the p.d.f. of R [11].

R is the generating variate, $g_{R}$ is the density generator and Σ is the scalar matrix. The elliptical vector *X* is unimodal if and only if its density generator $g_{R}$ is monotonically non-increasing. Most elliptical random vectors are unimodal, including multivariate normal distribution, multivariate T-distribution, multivariate Laplace distribution, and many others [12].

The elliptical distribution family has great properties, including summation stability, which ensures the sum of independent elliptically distributed random vectors with the same covariance generating matrix is elliptical [13]. The elliptical distribution family is broadly used in statistics studies. Many findings related to the normal distribution perform well when applied to the elliptical distribution. Based on the assumption of elliptical distribution, we develop the ROC curve function of likelihood ratio combination. Additionally, we derived the estimation of the elliptical likelihood ratio combination.

## 2. The Combination of Biomarkers Based on Elliptical Distribution

Elliptical distributions have several notable properties, such as summation stability (meaning the sum of independent elliptically distributed random vectors with the same covariance generating matrix remains elliptical). Another important property is infinite divisibility, allowing the distribution to be expressed as the sum of an arbitrary number of independent and identically distributed random variables. Additionally, elliptical distributions are self-decomposable. These properties make the elliptical distribution family particularly useful in various statistical applications, including robust statistics and generalized multivariate analysis. Given that many statistical techniques developed for the normal distribution also perform well when extended to elliptical distributions, it is both practical and feasible to extend the optimal combination methodology from the multivariate normal distribution to the broader elliptical family.

### 2.1. Likelihood Ratio Combination of Biomarkers with Elliptical Distribution

**Proposition** **1.** *Suppose there are d-dimensional biomarkers which are elliptically distributed, denoted as $X∼E_{d}\left(\mu_{x},\Sigma_{x};\phi_{x}\right)$ and $Y∼E_{d}\left(\mu_{y},\Sigma_{y};\phi_{y}\right)$ in case and control groups, respectively. The vectors X,Y could be stochastically represented as $X\overset{d}{=}\mu_{x}+R_{x}\Lambda_{x}U_{x}^{\left(d\right)}$ and $Y\overset{d}{=}\mu_{y}+R_{y}\Lambda_{y}U_{y}^{\left(d\right)}$ where $\Lambda_{x}\Lambda_{x}^{T}=\Sigma_{x}$ and $\Lambda_{y}\Lambda_{y}^{T}=\Sigma_{y}$. Then, the likelihood ratio (LR) combination, which is the optimal combination, could be expressed as*

(3) $$R\left(M\right)=\frac{g_{R_{x}}\left({\left(M-\mu_{x}\right)}^{T}\Sigma_{x}^{-1}\left(M-\mu_{x}\right)\right)}{g_{R_{y}}\left({\left(M-\mu_{y}\right)}^{T}\Sigma_{y}^{-1}\left(M-\mu_{y}\right)\right)}$$

*where $g_{R_{x}}$ and $g_{R_{y}}$ are density generators of X and Y, respectively.*

A subject will be diagnosed as diseased if the likelihood ratio combination R(M) exceeds a certain threshold c∈R. Generally, obtaining a likelihood ratio combination is complicated. However, under the assumption of elliptical distributions, this calculation can be simplified to the ratio of the density generators.

**Example** **1.** *(Multivariate Normal Distribution) Suppose there are d biomarkers following multivariate normal distributions in the case and control group: $X∼MN_{d}\left(\mu_{x},\Sigma_{x}\right)$, $Y∼MN_{d}\left(\mu_{y},\Sigma_{y}\right)$.*

*The density generator of d-dimensional multivariate normal distribution is $g\left(t\right)=\frac{1}{{\left(2\pi \right)}^{d/2}}\cdot \exp\left(-\frac{t}{2}\right)$ [10]. Then, the likelihood ratio combination is:*

$$\begin{array}{cc} R(M) & =\frac{g_{R_{x}}\left({\left(M-\mu_{x}\right)}^{T}\Sigma_{x}^{-1}\left(M-\mu_{x}\right)\right)}{g_{R_{y}}\left({\left(M-\mu_{y}\right)}^{T}\Sigma_{y}^{-1}\left(M-\mu_{y}\right)\right)}\\ & =\exp\left\{-\frac{1}{2}\left({\left(M-\mu_{x}\right)}^{T}\Sigma_{x}^{-1}\left(M-\mu_{x}\right)-{\left(M-\mu_{y}\right)}^{T}\Sigma_{y}^{-1}\left(M-\mu_{y}\right)\right)\right\}\\ & \propto -\left({\left(M-\mu_{x}\right)}^{T}\Sigma_{x}^{-1}\left(M-\mu_{x}\right)-{\left(M-\mu_{y}\right)}^{T}\Sigma_{y}^{-1}\left(M-\mu_{y}\right)\right) \end{array}$$

*A subject M will be diagnosed as diseased if R(M)>c, which is equivalent to that $({\left(M-\mu_{x}\right)}^{T}\Sigma_{x}^{-1}$$\left(M-\mu_{x}\right)-{\left(M-\mu_{y}\right)}^{T}\Sigma_{y}^{-1}\left(M-\mu_{y}\right))$ less than a cut point value. In particular, when $\Sigma_{x}=\Sigma_{y}=\Sigma$, the likelihood ratio becomes:*

$$\begin{array}{cc} R(M) & =\exp\left\{{\left(\mu_{x}-\mu_{y}\right)}^{T}\Sigma^{-1}M-\frac{1}{2}\left(\mu_{x}^{T}\Sigma^{-1}\mu_{x}-\mu_{y}^{T}\Sigma^{-1}\mu_{y}\right)\right\}\\ & \propto {\left(\mu_{x}-\mu_{y}\right)}^{T}\Sigma^{-1}M \end{array}$$

*A subject M will be diagnosed as diseased if R(M)>c, which is equivalent to that ${\left(\mu_{x}-\mu_{y}\right)}^{T}\Sigma^{-1}M$ is larger than a cut point value. Under this condition, the likelihood ratio combination is a linear combination with linear coefficient $\lambda =\Sigma^{-1}\left(\mu_{x}-\mu_{y}\right)$ [6].*

**Example** **2.** *(Multivariate T Distribution) Suppose there are d biomarkers following multivariate T distributions in the case and control group: $X∼t_{d}\left(\mu_{x},\Sigma ,v_{x}\right)$ and $Y∼t_{d}\left(\mu_{y},\Sigma ,v_{y}\right)$.*

*The density generator of multivariate t distribution $t_{d}\left(\mu ,\Sigma ,v\right)$ is $g_{R}\left(t\right)=\frac{\Gamma \left(\frac{d+v}{2}\right)}{\Gamma \left(\frac{v}{2}\right)}\cdot \frac{1}{{\left(v\pi \right)}^{d/2}}\cdot {\left(1+\frac{t}{v}\right)}^{-\frac{d+v}{2}}$ [10]. Then, the likelihood ratio combination is:*

$$\begin{array}{cc} R(M) & =\frac{g_{R_{x}}\left({\left(M-\mu_{x}\right)}^{T}\Sigma_{x}^{-1}\left(M-\mu_{x}\right)\right)}{g_{R_{y}}\left({\left(M-\mu_{y}\right)}^{T}\Sigma_{y}^{-1}\left(M-\mu_{y}\right)\right)}\\ & \propto {\left(1+\frac{{\left(M-\mu_{x}\right)}^{T}\Sigma_{x}^{-1}\left(M-\mu_{x}\right)}{v_{x}}\right)}^{-\frac{d+v_{x}}{2}}/{\left(1+\frac{{\left(M-\mu_{y}\right)}^{T}\Sigma_{y}^{-1}\left(M-\mu_{y}\right)}{v_{y}}\right)}^{-\frac{d+v_{y}}{2}}\\ & \propto \frac{{\left(v_{x}+{\left(M-\mu_{x}\right)}^{T}\Sigma_{x}^{-1}\left(M-\mu_{x}\right)\right)}^{-\frac{d+v_{x}}{2}}}{{\left(v_{y}+{\left(M-\mu_{y}\right)}^{T}\Sigma_{y}^{-1}\left(M-\mu_{y}\right)\right)}^{-\frac{d+v_{y}}{2}}} \end{array}$$

*In particular, if $v_{x}=v_{y}=v$, i.e., the degree freedoms of the case and control group are equal, the likelihood ratio combination is given by:*

$$R\left(M\right)={\left(\frac{v+{\left(M-\mu_{x}\right)}^{T}\Sigma_{x}^{-1}\left(M-\mu_{x}\right)}{v+{\left(M-\mu_{y}\right)}^{T}\Sigma_{y}^{-1}\left(M-\mu_{y}\right)}\right)}^{-\frac{d+v}{2}}$$

*Under this condition, A subject M will be diagnosed as diseased if the value of $\frac{v+{\left(M-\mu_{y}\right)}^{T}\Sigma_{y}^{-1}\left(M-\mu_{y}\right)}{v+{\left(M-\mu_{x}\right)}^{T}\Sigma_{x}^{-1}\left(M-\mu_{x}\right)}$ is larger than a cut point c.*

**Example** **3.** *(Mixed Distribution) Suppose $X∼MN_{d}\left(\mu_{x},\Sigma_{x}\right)$ and $Y∼t_{d}\left(\mu_{y},\Sigma_{y},v\right)$. Here, the distributions in the case and control group have different distribution “types”.*

*Then the likelihood ratio combination is:*

$$\begin{array}{cc} R(M) & =\frac{g_{R_{x}}\left({\left(M-\mu_{x}\right)}^{T}\Sigma_{x}^{-1}\left(M-\mu_{x}\right)\right)}{g_{R_{y}}\left({\left(M-\mu_{y}\right)}^{T}\Sigma_{y}^{-1}\left(M-\mu_{y}\right)\right)}\\ & \propto \exp\left(-\frac{1}{2}{\left(M-\mu_{x}\right)}^{T}\Sigma_{x}^{-1}\left(M-\mu_{x}\right)\right){\left(1+\frac{{\left(M-\mu_{y}\right)}^{T}\Sigma_{y}^{-1}\left(M-\mu_{y}\right)}{v}\right)}^{\frac{d+v}{2}}\\ & \propto \left(d+v\right)\log\left(v+{\left(M-\mu_{y}\right)}^{T}\Sigma_{y}^{-1}\left(M-\mu_{y}\right)\right)-{\left(M-\mu_{x}\right)}^{T}\Sigma_{x}^{-1}\left(M-\mu_{x}\right) \end{array}$$

### 2.2. Linear Combination of Biomarkers with Elliptical Distribution

Consider the linear combination of *d* biomarkers. Let $l\left(M\right)=\lambda^{T}M$ where $\lambda ={\left(\lambda_{1},\lambda_{2},\ldots ,\lambda_{d}\right)}^{T}$ is the combination coefficients. A subject will be diagnosed as diseased if l(M) is larger than a cut point. In 1993, Su and Liu derived the linear combined ROC curve under multivariate normal distribution. If $X∼N_{d}\left(\mu_{x},\Sigma_{x}\right)$ and $Y∼N_{d}\left(\mu_{y},\Sigma_{y}\right)$ are distribution of case and control group, respectively, given any specificity *p*, the corresponding sensitivity *s* could be represented by $S\left(p,\lambda \right)=\Phi \left(\frac{\lambda^{T}\left(\mu_{x}-\mu_{y}\right)-\Phi^{-1}\left(p\right)\sqrt{\lambda^{T}\Sigma_{x}\lambda }}{\sqrt{\lambda^{T}\Sigma_{y}\lambda }}\right)$, where λ is the linear combination coefficient [6]. Here we obtained the linear combined ROC curve based on elliptical distribution.

**Proposition** **2.** *Suppose $X∼E_{d}\left(\mu_{x},\Sigma_{x};\phi_{x}\right)$ and $Y∼E_{d}\left(\mu_{y},\Sigma_{y};\phi_{y}\right)$ are distribution of case and control group, respectively, then the linear combined ROC curve could be expressed as*

(4) $$s\left(t,\lambda \right)=F_{0}\left(\frac{\lambda^{T}\left(\mu_{x}-\mu_{y}\right)}{\sqrt{\lambda^{T}\Sigma_{x}\lambda }}-\frac{\sqrt{\lambda^{T}\Sigma_{y}\lambda }}{\sqrt{\lambda^{T}\Sigma_{x}\lambda }}G_{0}^{-1}\left(1-t\right)\right)$$

*where s is the sensitivity (TPR) and t is the 1-specificity (FPR). $F_{0}$ is the c.d.f of $\frac{\lambda^{T}X-\lambda^{T}\mu_{x}}{\lambda^{T}\Sigma_{x}\lambda }$ and $G_{0}$ is the c.d.f of $\frac{\lambda^{T}Y-\lambda^{T}\mu_{y}}{\lambda^{T}\Sigma_{y}\lambda }$*

**Proof.**  Let $X_{0}=\frac{X-\lambda^{T}\mu_{x}}{\lambda^{T}\Sigma_{x}\lambda }∼E_{1}\left(0,1;\phi_{x}\right)$ with p.d.f $f_{0}$ and c.d.f $F_{0}$, $Y_{0}=\frac{Y-\lambda^{T}\mu_{y}}{\lambda^{T}\Sigma_{y}\lambda }∼E_{1}\left(0,1;\phi_{y}\right)$ with p.d.f $g_{0}$ and c.d.f $G_{0}$. Since $f_{0}$, $g_{0}$ are both symmetric functions, we have $f_{0}\left(x\right)=f_{0}\left(-x\right)$, $g_{0}\left(y\right)=g_{0}\left(-y\right)$, $1-F_{0}\left(x\right)=F_{0}\left(-x\right)$ and $1-G_{0}\left(y\right)=G_{0}\left(-y\right)$.

For each cut point −∞<c<∞, the false positive rate (1-specificity) *t* could be represented by:

$$t=P\left(Y>c\right)=P\left(\frac{Y-\lambda^{T}\mu_{y}}{\sqrt{\lambda^{T}\Sigma_{y}\lambda }}>\frac{c-\lambda^{T}\mu_{y}}{\sqrt{\lambda^{T}\Sigma_{y}\lambda }}\right)=G_{0}\left(\frac{\lambda^{T}\mu_{y}-c}{\sqrt{\lambda^{T}\Sigma_{y}\lambda }}\right)$$

So $c=\lambda^{T}\mu_{y}-\sqrt{\lambda^{T}\Sigma_{y}\lambda }G_{0}^{-1}\left(t\right)$. Then the sensitivity *s* is given by:

$$\begin{array}{cc} s(t,\lambda ) & =P(X>c)\\ & =F_{0}\left(\frac{\lambda^{T}\mu_{x}-c}{\sqrt{\lambda^{T}\Sigma_{x}\lambda }}\right)\\ & =F_{0}\left(\frac{\lambda^{T}\mu_{x}-\lambda^{T}\mu_{y}+\sqrt{\lambda^{T}\Sigma_{y}\lambda }G_{0}^{-1}\left(t\right)}{\sqrt{\lambda^{T}\Sigma_{x}\lambda }}\right)\\ & =F_{0}\left(\frac{\lambda^{T}\left(\mu_{x}-\mu_{y}\right)}{\sqrt{\lambda^{T}\Sigma_{x}\lambda }}-\frac{\sqrt{\lambda^{T}\Sigma_{y}\lambda }}{\sqrt{\lambda^{T}\Sigma_{x}\lambda }}G_{0}^{-1}\left(1-t\right)\right) \end{array}$$

□

In particular, when $\Sigma_{x}=\Sigma_{y}=\Sigma$, the ROC curve function could be simplified to $s\left(t,\lambda \right)=F_{0}\left(\frac{\lambda^{T}\left(\mu_{x}-\mu_{y}\right)}{\sqrt{\lambda^{T}\Sigma \lambda }}-G_{0}^{-1}\left(1-t\right)\right)$.

## 3. Non-Parametric Estimation of Elliptical Combined ROC Curve

The non-parametric maximum likelihood estimate (NPMLE) of the density generator for the elliptical unimodal distribution was derived by [12]. Sun et al. proposed parameters estimation of a multivariate elliptical distribution to fit data via Tyler’s method [14]. Building upon these, we propose the empirical estimation of ROC curve functions for the likelihood ratio combinations in an elliptical distribution.

Suppose there are *n* independent observations $\left(X_{1},\;X_{2},\;\cdots ,X_{n}\right)∼E_{d}\left(\mu_{x},\Sigma_{x};\phi_{x}\right)$ in case group, and *m* independent observations $\left(Y_{1},\;Y_{2},\;\cdots ,Y_{m}\right)∼E_{d}\left(\mu_{y},\Sigma_{y};\phi_{y}\right)$ in control group. The density generators in both groups are monotonically non-increasing, which means these biomarkers are elliptical and unimodally distributed.

The following are the main steps to obtain the empirical estimation of the likelihood ratio combination ROC curve:

Step 1:  Define ${\hat{\mu }}_{x}$ and ${\hat{\Sigma }}_{x}$ as the mean vector and scatter matrix estimates, respectively, obtained for the case group using Tyler’s method [14]. Similarly, let ${\hat{\mu }}_{y}$ and ${\hat{\Sigma }}_{y}$ denote the mean vector and scatter matrix estimates for the control group observations.
Step 2:  Let $\left(Z_{\left(1\right)},\ldots ,Z_{\left(n\right)}\right)$ be the order statistics of $\left(Z_{1},\;Z_{2},\;\cdots ,\;Z_{n}\right)$, where $Z_{i}={\left(X_{i}-{\hat{\mu }}_{x}\right)}^{T}\Sigma^{-1}\left(X_{i}-{\hat{\mu }}_{x}\right)$, i∈(1,2,⋯,n).
Step 3:  Define
  (5) $${\hat{g}}_{i}=\frac{d}{2c_{d}}\min_{s\leq i-1}\max_{t\geq i}\frac{F_{n}\left(Z_{\left(t\right)}\right)-F_{n}\left(Z_{\left(s\right)}\right)}{Z_{\left(t\right)}^{d/2}-Z_{\left(s\right)}^{d/2}}$$
  where $F_{n}$ is the empirical cumulative distribution function (CDF) of the data $\left(Z_{1},\ldots ,Z_{n}\right)$, $c_{d}=\frac{\pi^{d/2}}{\Gamma (d/2)}$ and $z\in R^{+}$.
Step 4:  ${\hat{g}}_{x}\left(z\right)=\left\{\begin{array}{cc} {\hat{g}}_{i}, & \;\mathrm{if}\;Z_{\left(i-1\right)}<z\leq Z_{\left(i\right)}\\ 0, & \;\mathrm{otherwise}\; \end{array}\right.$ is the non-parametric maximum likelihood estimate (NPMLE) of density generator $g_{R_{x}}$.
Step 5:  Similarly, ${\hat{g}}_{y}$, the NPMLE of the density generator $g_{R_{y}}$, can be obtained by repeating steps 2 to 4 for the control group observations.
Step 6:  For i∈(1,2,⋯,n), let ${\hat{R}}_{i}\left(X\right)=\frac{{\hat{g}}_{x}\left({\left(X_{i}-{\hat{\mu }}_{x}\right)}^{T}\Sigma_{x}^{-1}\left(X_{i}-{\hat{\mu }}_{x}\right)\right)}{{\hat{g}}_{y}\left({\left(X_{i}-{\hat{\mu }}_{y}\right)}^{T}\Sigma_{y}^{-1}\left(X_{i}-{\hat{\mu }}_{y}\right)\right)}$.
Step 7:  For j∈(1,2,⋯,m), let ${\hat{R}}_{j}\left(Y\right)=\frac{{\hat{g}}_{x}\left({\left(Y_{j}-{\hat{\mu }}_{x}\right)}^{T}\Sigma_{x}^{-1}\left(Y_{j}-{\hat{\mu }}_{x}\right)\right)}{{\hat{g}}_{y}\left({\left(Y_{j}-{\hat{\mu }}_{y}\right)}^{T}\Sigma_{y}^{-1}\left(Y_{j}-{\hat{\mu }}_{y}\right)\right)}$.
Step 8:  For a cut point value c∈R, Let $\hat{s}\left(c\right)=\frac{\sum_{i=1}^{n}I\left({\hat{R}}_{i}\left(X\right)>c\right)}{n}$, $\hat{p}\left(c\right)=\frac{\sum_{j=1}^{m}I\left({\hat{R}}_{j}\left(Y\right)>c\right)}{m}$, where *I* is the indicator function.
Step 9:  The estimated ROC curve of likelihood ration combination is obtained by plotting $\hat{s}\left(c\right)$ and $1-\hat{p}\left(c\right)$ over all values of c∈R.

## 4. Simulation

To evaluate the performance of the estimation method described above, we conducted simulations comparing the AUC values obtained using three different combinations: the proposed elliptical combination, optimal normal combination, and best linear combination. We assume that biomarkers in the case and control groups follow a multivariate gamma distribution:

$$X∼MG\left(s_{x},r_{x},\Sigma_{x}\right),\;Y∼MG\left(s_{y},r_{y},\Sigma_{y}\right)$$

Here, $s_{x}$ is a vector of shape parameters for the marginal distributions of the columns of *X*, $r_{x}$ is a vector of rate parameters for the marginal distributions of the columns of *X*, and $\Sigma_{x}$ is the correlation matrix. Similarly, $s_{y}$, $r_{y}$, and $\Sigma_{y}$ represent the parameters for the control group biomarkers

We chose the multivariate gamma distribution for simulation for several reasons. First, multivariate gamma variables inherently take values greater than 0, which aligns with the typical range of biomarker values encountered in practical scenarios. Additionally, this distribution allows flexibility in adjusting the shape parameters *s* and rate parameters *r*, thereby enabling us to model both symmetric and skewed distributions as needed. This choice of distribution facilitates a realistic simulation environment that reflects the characteristics observed in actual biomarker data.

### 4.1. Both X and Y are Approximately Symmetric

Firstly, we begin with the condition of approximately symmetric distributions. Suppose there are four biomarkers, let $s_{x}=\left(\begin{array}{c} 20\\ 13\\ 20\\ 12 \end{array}\right),\;s_{y}=\left(\begin{array}{c} 15\\ 13\\ 10\\ 20 \end{array}\right),\;r_{x}=\left(\begin{array}{c} 5\\ 1\\ 2\\ 1 \end{array}\right),\;r_{y}=\left(\begin{array}{c} 3\\ 1\\ 1.2\\ 1 \end{array}\right)$

$\Sigma_{x}=\left(\begin{array}{cccc} 1 & 0.2 & 0.3 & 0.1\\ 0.2 & 2 & 0.2 & 0.02\\ 0.3 & 0.2 & 1 & 0.1\\ 0.1 & 0.02 & 0.1 & 2 \end{array}\right)$ and $\Sigma_{y}=\left(\begin{array}{cccc} 1 & 0.1 & 0.1 & 0.6\\ 0.1 & 1.5 & 0.2 & 0.15\\ 0.1 & 0.2 & 1 & 0.6\\ 0.6 & 0.15 & 0.6 & 1 \end{array}\right)$. 

**Figure 1:**
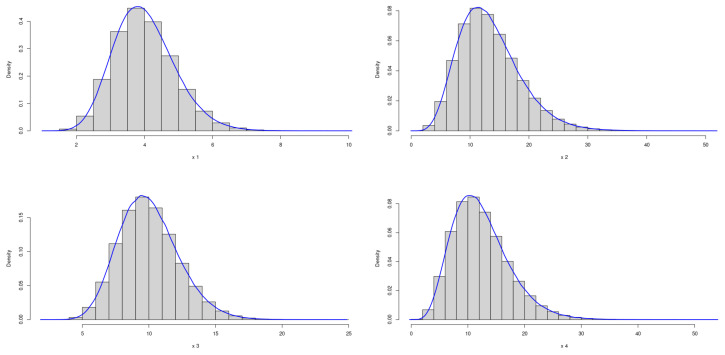
Marginal Distributions of *X*.

**Figure 2:**
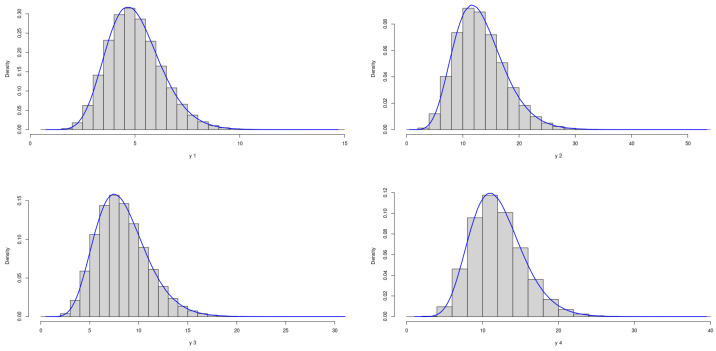
Marginal Distributions of *Y*.

The theoretical optimal AUC is 0.90. Figure 1 and Figure 2 demonstrate the marginal distributions of these biomarkers in the case and control group, respectively. In either the case or control group, the marginal distributions of four biomarkers are nearly symmetric.

Based on Pepe et al. (2006), the optimal combination is the likelihood ratio combination:

(6) $$\begin{array}{cc} \; & R\left(M\right)=\frac{f_{X}\left(M_{1},\ldots ,M_{4}\right)}{f_{Y}\left(M_{1},\ldots ,M_{4}\right)} \end{array}$$

where $f_{X}$ and $f_{Y}$ are density functions for case and control group, respectively [9].

We generated $n_{x}$ observations in the case group and $n_{y}$ observations in the control group, with $n_{x}<n_{y}$, which is typically the case in practice. We can evaluate the results for different sample sizes of $n_{x}$ and $n_{y}$. The AUC was calculated using the proposed elliptical combination, multivariate normal combination, and linear combination methods. The optimal combination can be determined using Formula (6), with the corresponding AUC representing the true optimal AUC. This true AUC was used to evaluate the performance of the three methods. Bias, defined as the difference between the estimated AUC and the true AUC, was used as a measure of performance, with a bias closer to 0 indicating better performance.

We repeated the above process 500 times and calculate the median and quantiles of the biases for the three methods. Table 1 presents the simulation results for different sample sizes in the case and control groups:

From the simulation results of the median bias, we observe that the proposed elliptical combination generally outperforms the multivariate normal combination and the linear combination when the sample size is large. However, as the sample size decreases, the multivariate normal combination tends to perform better than the elliptical estimation, indicating that the Non-parametric Maximum Likelihood Estimator (NPMLE) for the elliptical combination requires a relatively large sample size to achieve optimal performance.

Overall, both the elliptical and normal combinations have better AUC values than the linear combination when all biomarkers exhibit approximately symmetric distributions in both the case and control groups. However, the elliptical combination is superior when we have a sufficient sample size, making it the preferred method in scenarios with larger datasets.

### 4.2. Both X and Y Are Skewed

Now, we consider the condition where all biomarkers are skewed in both the case and control groups. Let $s_{x}=\left(\begin{array}{c} 2.5\\ 2.8\\ 3\\ 5 \end{array}\right),\;s_{y}=\left(\begin{array}{c} 3\\ 2\\ 3.5\\ 3 \end{array}\right),\;r_{x}=\left(\begin{array}{c} 1.5\\ 1\\ 3\\ 1 \end{array}\right),\;r_{y}=\left(\begin{array}{c} 2\\ 2\\ 3\\ 0.6 \end{array}\right)$

$\Sigma_{x}=\left(\begin{array}{cccc} 1.5 & 0.2 & 0.3 & 0.1\\ 0.2 & 2 & 0.2 & 0.02\\ 0.3 & 0.2 & 1 & 0.1\\ 0.1 & 0.02 & 0.1 & 2 \end{array}\right)$ and $\Sigma_{y}=\left(\begin{array}{cccc} 1.4 & 0.1 & 0.1 & 0.6\\ 0.1 & 1.5 & 0.2 & 0.15\\ 0.1 & 0.2 & 1 & 0.6\\ 0.6 & 0.15 & 0.6 & 1 \end{array}\right)$. 

**Figure 3:**
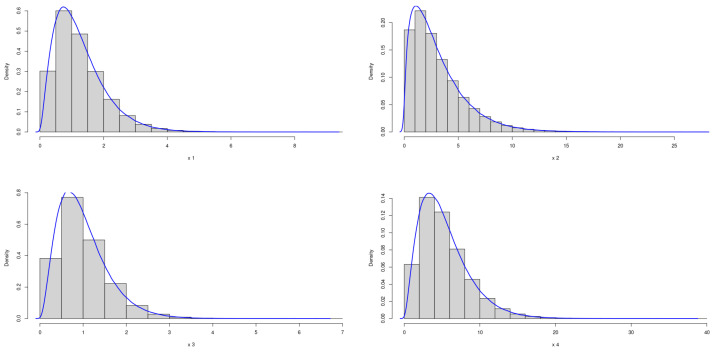
Marginal Distributions of *X*.

**Figure 4:**
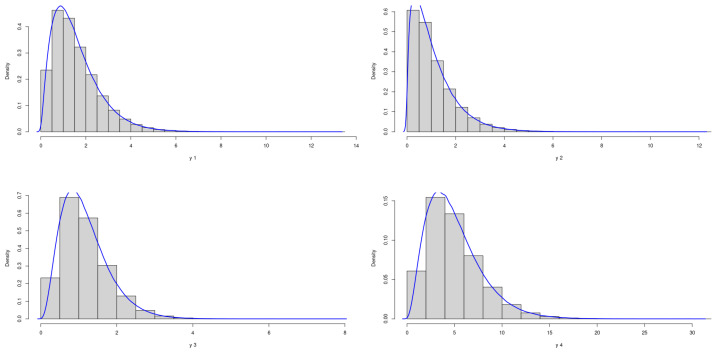
Marginal Distributions of *Y*.

The theoretical optimal AUC is 0.885. Figure 3 and Figure 4 demonstrate the marginal distributions of these biomarkers in the case and control group, respectively. We can see that in both the case and control groups, the marginal distributions of four biomarkers are skewed.

Using the same simulation process as before, we generated the results under the condition that all biomarkers are skewed in both the case and control groups. Below are the results for different sample sizes of $n_{x}$ and $n_{y}$:

From the simulation results in Table 2, it is evident that both the elliptical and multivariate normal combinations perform obviously better than the linear combination across all conditions. Furthermore, the proposed elliptical combination consistently outperforms the multivariate normal combination. For all sample sizes, the medians of biases for the elliptical combination are always closer to 0 than those of the multivariate normal combination. Additionally, the interquartile ranges (IQRs) of the elliptical combination consistently cover 0, indicating balanced performance across sample sizes, while the IQRs of the multivariate normal combination are always less than 0, suggesting a tendency to underestimate the AUC.

Notably, the elliptical combination shows superior performance over the multivariate normal and linear combinations when both the case and control groups exhibit skewed distributions. This makes the elliptical combination particularly advantageous in scenarios involving skewed biomarker distributions, as it provides more accurate and reliable AUC estimates.

### 4.3. X is Approximately Symmetric and Y is Skewed

Next, we consider the condition where one of the case and control groups is skewed, and the other is nearly symmetric. There is no difference in which group is skewed. For simplicity, we assume the case group *X* is near symmetric while the control group *Y* is skewed.

let $s_{x}=\left(\begin{array}{c} 10\\ 14\\ 21\\ 14 \end{array}\right),\;s_{y}=\left(\begin{array}{c} 2.4\\ 2\\ 2.5\\ 2 \end{array}\right),\;r_{x}=\left(\begin{array}{c} 5.2\\ 6.7\\ 7.9\\ 5 \end{array}\right),\;r_{y}=\left(\begin{array}{c} 1.2\\ 1\\ 1\\ 0.8 \end{array}\right)$

$\Sigma_{x}=\left(\begin{array}{cccc} 2 & 0.2 & 0.3 & 0.1\\ 0.2 & 2.1 & 0.2 & 0.02\\ 0.3 & 0.2 & 1.5 & 0.1\\ 0.1 & 0.02 & 0.1 & 2.3 \end{array}\right)$ and $\Sigma_{y}=\left(\begin{array}{cccc} 1 & 0.1 & 0.1 & 0.6\\ 0.1 & 1 & 0.2 & 0.15\\ 0.1 & 0.2 & 1.6 & 0.6\\ 0.6 & 0.15 & 0.6 & 1 \end{array}\right)$. 

**Figure 5:**
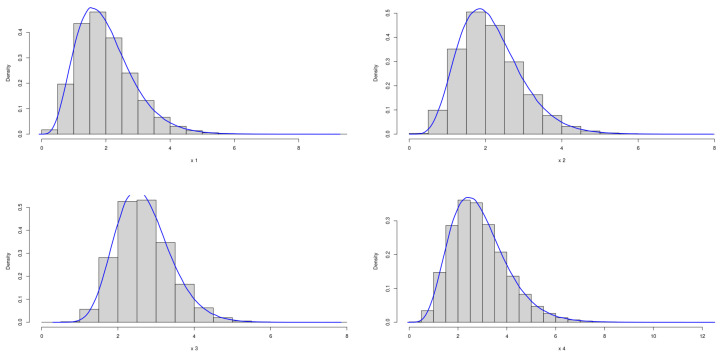
Marginal Distributions of *X*.

**Figure 6:**
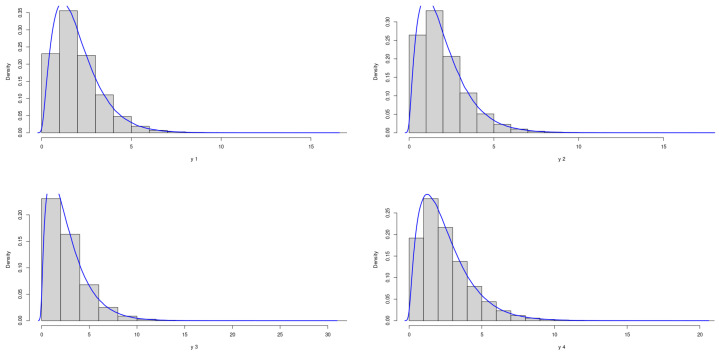
Marginal Distributions of *Y*.

The theoretical optimal AUC is 0.899. Figure 5 and Figure 6 demonstrate the marginal distributions of these biomarkers in the case and control group, respectively. Using the same simulation process as before, Table 3 presents the results across various sample sizes:

The simulation results demonstrate that the proposed elliptical combination consistently performs better than both the multivariate normal and linear combinations. Across all sample sizes, the elliptical combination exhibits medians of biases that are consistently closer to 0 compared to the other methods.

Additionally, the IQRs of the elliptical combination reliably encompass 0, reflecting a more balanced estimation. In contrast, the IQRs of the multivariate normal and linear combinations are persistently negative, indicating a systematic tendency to underestimate the AUC.

These findings underscore the effectiveness of the elliptical combination in situations where one group exhibits skewness while the other remains symmetric. This approach proves to be more reliable and accurate in estimating the AUC compared to the multivariate normal and linear combinations, making it a preferred method in such scenarios.

**Table 3:** Biases of Combined AUC values for Different Methods when *X* is Near Symmetric and *Y* is Skewed.

Bias of Estimated AUC				
		**Elliptical Combination**	**Multi-Normal Combination**	**Linear Combination**
$n_{x}=500$ $n_{y}=700$	Median	−0.0066	−0.0148	−0.2670
	Q1, Q3	−0.0133, 0.0002	−0.0203, −0.0096	−0.2913, −0.2391
	Min, Max	−0.0257, 0.0064	−0.0272, −0.0063	−0.3142, −0.2202
$n_{x}=375$ $n_{y}=525$	Median	−0.0039	−0.0139	−0.2658
	Q1, Q3	−0.0125, 0.0043	−0.0208, −0.0075	−0.2975, −0.2340
	Min, Max	−0.0175, 0.0113	−0.0253, −0.0024	−0.3141, −0.2214
$n_{x}=300$ $n_{y}=500$	Median	−0.0029	−0.0137	−0.2656
	Q1, Q3	−0.0108, 0.0053	−0.0206, −0.0071	−0.2969, −0.2351
	Min, Max	−0.0189, 0.0135	−0.0250, 0.0012	−0.3196, −0.1956
$n_{x}=250$ $n_{y}=350$	Median	−0.0005	−0.0127	−0.2628
	Q1, Q3	−0.0115, 0.0099	−0.0210, −0.0049	−0.3017, −0.2273
	Min, Max	−0.0258, 0.0172	−0.0288, 0.0025	−0.3339, −0.1849
$n_{x}=200$ $n_{y}=280$	Median	0.0027	−0.0122	−0.2593
	Q1, Q3	−0.0097, 0.0139	−0.0223, −0.0031	−0.3012, −0.2221
	Min, Max	−0.0195, 0.0273	−0.0331, 0.0039	−0.3521, −0.1863

### 4.4. Mixed Distributions in X and Y: Approximately Symmetric and Skewed

Lastly, we consider the condition where the biomarkers exhibit a combination of skewed and symmetric distributions in both the case and control groups. 

let $s_{x}=\left(\begin{array}{c} 2\\ 4\\ 3\\ 1 \end{array}\right),\;s_{y}=\left(\begin{array}{c} 3\\ 2\\ 4\\ 1 \end{array}\right),\;r_{x}=\left(\begin{array}{c} 0.3\\ 2\\ 0.2\\ 1 \end{array}\right),\;r_{y}=\left(\begin{array}{c} 0.5\\ 0.7\\ 0.2\\ 0.5 \end{array}\right)$

$\Sigma_{x}=\left(\begin{array}{cccc} 0.5 & 0.2 & 0.3 & 0.1\\ 0.2 & 2 & 0.2 & 0.02\\ 0.3 & 0.2 & 1 & 0.1\\ 0.1 & 0.02 & 0.1 & 2 \end{array}\right)$ and $\Sigma_{y}=\left(\begin{array}{cccc} 1 & 0.1 & 0.1 & 0.6\\ 0.1 & 1.5 & 0.2 & 0.15\\ 0.1 & 0.2 & 1 & 0.6\\ 0.6 & 0.15 & 0.6 & 2 \end{array}\right)$. 

**Figure 7:**
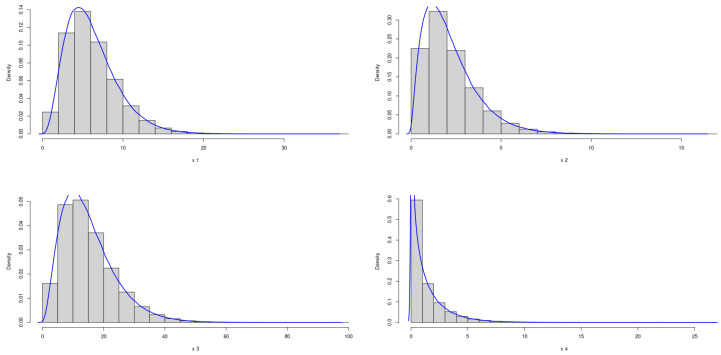
Marginal Distributions of *X*.

**Figure 8:**
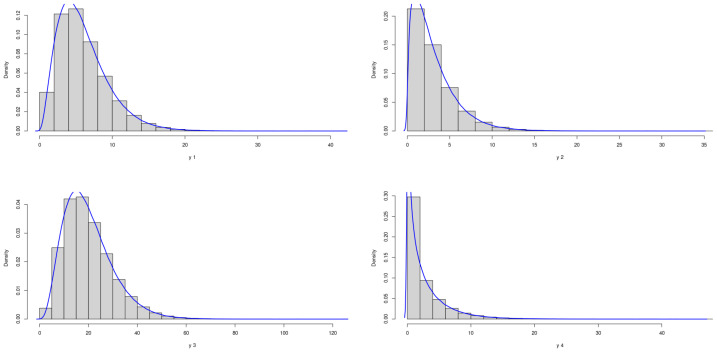
Marginal Distributions of *Y*.

The theoretical optimal AUC is 0.785. Figure 7 and Figure 8 demonstrate the marginal distributions of these biomarkers in the case and control group, respectively. The second and fourth biomarkers in both groups are skewed, while the first and third biomarkers are more symmetric.

The simulation results in the Table 4 indicate that the proposed elliptical combination performs better than both the multivariate normal combination and the linear combination when the biomarkers display a mixture of skewed and symmetric distributions in both the case and control groups. Although the multivariate normal and linear methods tend to produce negative biases, signaling a propensity to underestimate, the elliptical combination maintains IQRs that include 0. This suggests that the elliptical method provides a more accurate and stable estimation of the AUC.

**Table 4:** Biases of Combined AUC values for Different Methods when Mixed Distributions in *X* and *Y*.

Bias of Estimated AUC				
		**Elliptical Combination**	**Multi-Normal Combination**	**Linear Combination**
$n_{x}=500$ $n_{y}=700$	Median	−0.0147	−0.0369	−0.0997
	Q1, Q3	−0.0248, 0.0037	−0.0496, −0.0254	−0.1333, −0.0648
	Min, Max	−0.0403, 0.0073	−0.0661, −0.0115	−0.2701, −0.0390
$n_{x}=375$ $n_{y}=525$	Median	−0.0097	−0.0358	−0.1021
	Q1, Q3	−0.0223, 0.0037	−0.0492, −0.0215	−0.1354, −0.0633
	Min, Max	−0.0295, 0.0186	−0.0602, −0.0073	−0.3415, −0.0355
$n_{x}=300$ $n_{y}=500$	Median	−0.0068	−0.0354	−0.1030
	Q1, Q3	−0.0201, 0.0077	−0.0507, −0.0196	−0.1392, −0.0659
	Min, Max	−0.0411, 0.0199	−0.0717, −0.0108	−0.3408, −0.0434
$n_{x}=250$ $n_{y}=350$	Median	−0.0010	−0.0343	−0.1026
	Q1, Q3	−0.0204, 0.0170	−0.0519, −0.0160	−0.1410, −0.0625
	Min, Max	−0.0330, 0.0347	−0.0866, −0.0015	−0.2508, −0.0359
$n_{x}=200$ $n_{y}=280$	Median	0.0053	−0.0316	−0.1051
	Q1, Q3	−0.0136, 0.0230	−0.0512, −0.0125	−0.1474, −0.0612
	Min, Max	−0.0308, 0.0320	−0.0603, 0.0018	−0.3638, −0.0425

Overall, the elliptical combination method consistently provided better performance across most conditions, demonstrating its robustness in handling various distribution types of biomarkers. The multivariate normal combination also performed well but showed tendencies to underestimate, particularly when dealing with skewed distributions. The linear combination was less effective, highlighting its limitations in comparison to the elliptical and multivariate normal methods. The proposed elliptical combination is more accurate and reliable in diverse conditions.

## 5. Application

We applied the proposed methods to case-control studies and compared the results with those from other established approaches. To evaluate the diagnostic performance of the biomarkers, we analyzed and compared the ROC curves and corresponding AUC values of the proposed elliptical likelihood ratio combination against two other methods: the multivariate normal combination proposed by Su and Liu [6] and the best linear combination introduced by Pepe et al. [9]. We expected that the proposed elliptical combination would yield a higher AUC than the alternatives. We began by demonstrating the application of the proposed combination method to the diagnosis of autism.

### 5.1. Biomarkers for Autism/Autistic Disorder

Autism/autism spectrum disorder (ASD) is a collection of developmental disorders characterized by challenges in communication, repetitive behaviors, and increased irritability [15]. The diagnosis of autism/autism spectrum disorders (ASD) has gained higher attention due to its diagnostic complexities. Failure to correctly diagnose children with autism/ASD may result in missed opportunities for appropriate treatments, while misdiagnosis of autism/ASD also leads to potentially harmful consequences for children [16].

In 2002–2005, the Eunice Kennedy Shriver National Institute of Child Health and Human Development conducted a case-control study to investigate the potential role of growth-related hormones in diagnosing autism in children aged 4 to 8 years [17]. The study included 59 subjects in the control group and 71 subjects in the case group. Each participant was tested for multiple diagnostic biomarkers, including insulin-like growth factors (IGF1, IGF2, IGFBP3 ), as well as dehydroepiandrosterone (DHEA) DHEA sulfate (DHEAS), and so on. Detailed descriptions of subject enrollment and data collection are provided by Mills et al. [17]. We will combine five biomarkers, including IGF1, IGF2, IGFBP3, DHEA, and DHEAS, from the data to diagnose autism/ASD and calculate combined AUC values.

**Figure 9:**
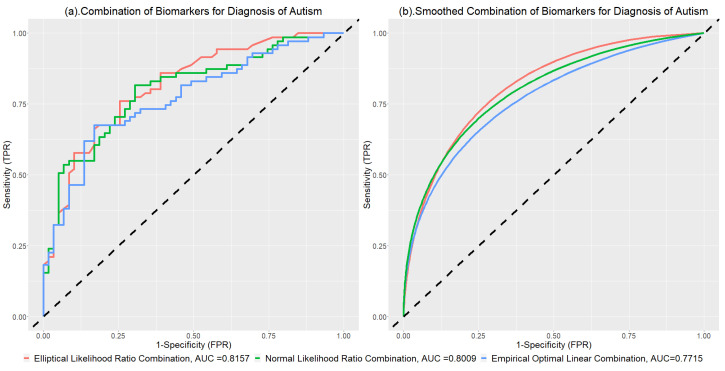
Combinations of Autism Data and Corresponding AUC Values.

The results of the application are presented in Figure 9a. Figure 9b shows the smoothed ROC curves for different combinations. The ROC curve of the elliptical likelihood ratio combination (red line) has the largest AUC value among all methods. Notably, the red line is obviously higher than all other ROC curves, particularly when the specificity is small. The multivariate normal likelihood ratio combination achieved an AUC value of 0.8009, which is higher than the empirical linear combination. The proposed elliptical combination further enhanced the AUC to 0.8157, demonstrating its superior ability to discriminate between case and control groups in this context.

### 5.2. Diagnosis of Neural Tube Defects (NTDs)

Neural tube defects (NTDs) are a group of birth defects, including spina bifida, anencephaly, and others [18]. NTDs are one of the most common birth defects. Maternal biomarkers play a crucial role in the early diagnosis and prevention of NTDs. By assessing the ROC curve and AUC values, we can gain insights into the potential biomarker combinations in diagnosing NTDs. This information is crucial for enhancing the accuracy and effectiveness of the screening program and providing valuable insights into the early detection and prevention of neural tube defects.

In the study conducted on the Irish National Rubella Screening Program, data were collected from blood samples obtained from pregnant women, with a focus on recording neural tube defects (NTDs). We chose four biomarkers related to the NTD diagnosis: vitamin B12, reduced red cell folate, plasma total folate, and free choline. After removing missing values, the dataset consisted of 71 observations in the case group (women with NTDs) and 230 observations in the control group (women without NTDs).

**Figure 10:**
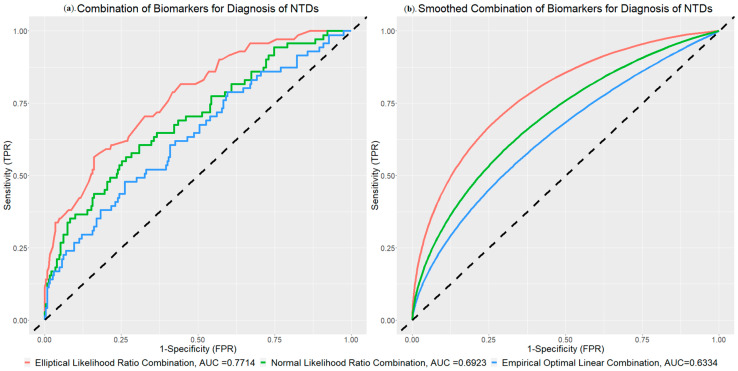
Combinations of NTDs Data and Corresponding AUC Values.

The results of the application are presented in Figure 10a. The multivariate normal likelihood ratio combination achieves an AUC value of 0.6923, which is slightly higher than the AUC value obtained with the best linear combinations. The proposed elliptical likelihood ratio combination exhibited an obviously improved AUC value of 0.7714. The elliptical likelihood ratio combination could improve the AUC by 11.43% and 21.79% compared to the multivariate normal likelihood ratio combination and best linear combination, respectively. This marked enhancement in performance demonstrated the effectiveness of utilizing elliptical distributions in the estimation process. In addition, the ROC curve of the elliptical likelihood ratio combination (represented by the red line) is consistently higher than all other combinations across various specificity values. Figure 10b shows the smoothed ROC curves for different combinations. Therefore, according to the combined ROC curves and AUC values, we can safely conclude that the elliptical combination significantly improved the diagnosis performance of biomarkers related to the NTDs.

## 6. Conclusions and Discussion

In this paper, we discuss the likelihood ratio combination of elliptically distributed biomarkers to enhance diagnostic accuracy. The non-parametric maximum likelihood estimation for the proposed method is developed. Simulation results show the advantages of using the elliptical combination for more accurate and reliable AUC calculations in diverse conditions. Compared to the multivariate normal combination, the elliptical likelihood ratio combination benefits from greater flexibility and robustness across a wider range of data distributions, including those with heavy or light tails, symmetric or skewed, making it more suitable for real-world applications. By relaxing the assumption of normality and allowing for elliptical distributions, we can fully leverage the potential of the biomarkers and improve their diagnostic accuracy.

We compare the performance of the normal likelihood ratio combination, the best linear combination, and the proposed elliptical likelihood ratio combination using autism/ASD and NTDs datasets. Our findings consistently demonstrate that the elliptical likelihood ratio combination outperforms the other combinations in terms of diagnostic accuracy.

## Figures and Tables

**Table 1:** Biases of Combined AUC Values for Different Methods when Both *X* and *Y* are Close to Symmetric Distributions.

Bias of Estimated AUC				
		**Elliptical Combination**	**Multi-Normal Combination**	**Linear Combination**
$n_{x}=500$ $n_{y}=700$	Median	0.0027	−0.0055	−0.0952
	Q1, Q3	−0.0020, 0.0074	−0.0095, −0.0013	−0.1273, −0.0736
	Min, Max	−0.0056, 0.0107	−0.0148, 0.0016	−0.1552, −0.0586
$n_{x}=375$ $n_{y}=525$	Median	0.0047	−0.0052	−0.0978
	Q1, Q3	−0.0013, 0.0100	−0.0105, 0.0003	−0.1323, −0.0717
	Min, Max	−0.0069, 0.0178	−0.0153, 0.0041	−0.1858, −0.0524
$n_{x}=300$ $n_{y}=500$	Median	0.0051	−0.0050	−0.0984
	Q1, Q3	−0.0007, 0.0115	−0.0101, −0.0001	−0.1377, −0.0724
	Min, Max	−0.0048, 0.0168	−0.0146, 0.0044	−0.1935, −0.0517
$n_{x}=250$ $n_{y}=350$	Median	0.0075	−0.0040	−0.1031
	Q1, Q3	0, 0.0148	−0.0101, 0.0022	−0.1434, −0.0712
	Min, Max	−0.0056, 0.0229	−0.0161, 0.0069	−0.1992, −0.0476
$n_{x}=200$ $n_{y}=280$	Median	0.0096	−0.0032	−0.1028
	Q1, Q3	0.0015, 0.0195	−0.0104, 0.0038	−0.1431, −0.0664
	Min, Max	−0.0109, 0.0283	−0.0144, 0.0102	−0.1901, −0.0370

**Table 2:** Biases of Combined AUC values for Different Methods when Both *X* and *Y* are Skewed.

Bias of Estimated AUC				
		**Elliptical Combination**	**Multi-Normal Combination**	**Linear Combination**
$n_{x}=500$ $n_{y}=700$	Median	−0.0084	−0.0188	−0.0449
	Q1, Q3	−0.0159, −0.0006	−0.0273, −0.0111	−0.0582, −0.0324
	Min, Max	−0.0247, 0.0046	−0.0353, −0.0028	−0.0695, −0.0246
$n_{x}=375$ $n_{y}=525$	Median	−0.0064	−0.0181	−0.0452
	Q1, Q3	−0.0150, 0.0019	−0.0286, −0.0093	−0.0608, −0.0309
	Min, Max	−0.0208, 0.0079	−0.0347, −0.0037	−0.0748, −0.0161
$n_{x}=300$ $n_{y}=500$	Median	−0.0052	−0.0181	−0.0444
	Q1, Q3	−0.0148, 0.0044	−0.0283, −0.0079	−0.0640, −0.0269
	Min, Max	−0.0225, 0.0145	−0.0368, 0.0031	−0.0792, −0.0168
$n_{x}=250$ $n_{y}=350$	Median	−0.0028	−0.0177	−0.0451
	Q1, Q3	−0.0136, 0.0080	−0.0298, −0.0061	−0.0650, −0.0268
	Min, Max	−0.0214, 0.0246	−0.0381, 0.0074	−0.0761, −0.0167
$n_{x}=200$ $n_{y}=280$	Median	0.0001	−0.0168	−0.0428
	Q1, Q3	−0.0128, 0.0128	−0.0295, −0.0032	−0.0701, −0.0229
	Min, Max	−0.0256, 0.0198	−0.0431, 0.0072	−0.0994, −0.0014

## Data Availability

The original contributions presented in the study are included in the article, further inquiries can be directed to the corresponding author.
